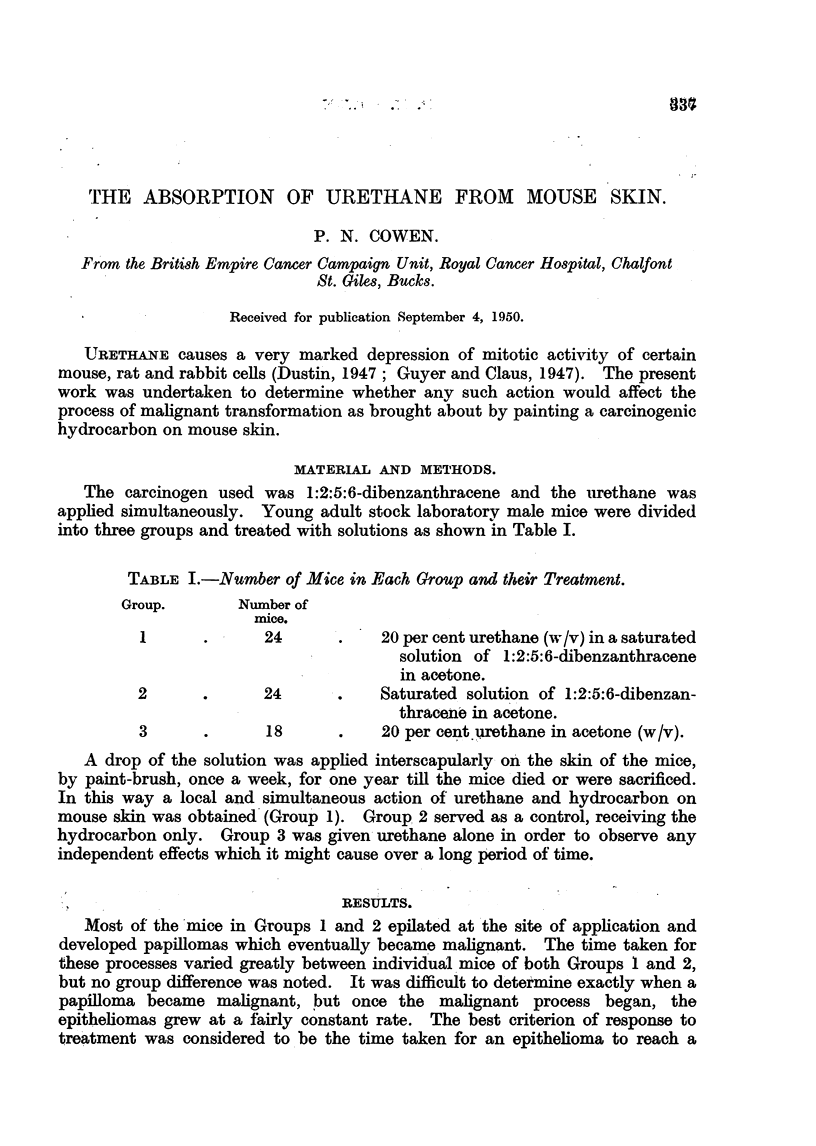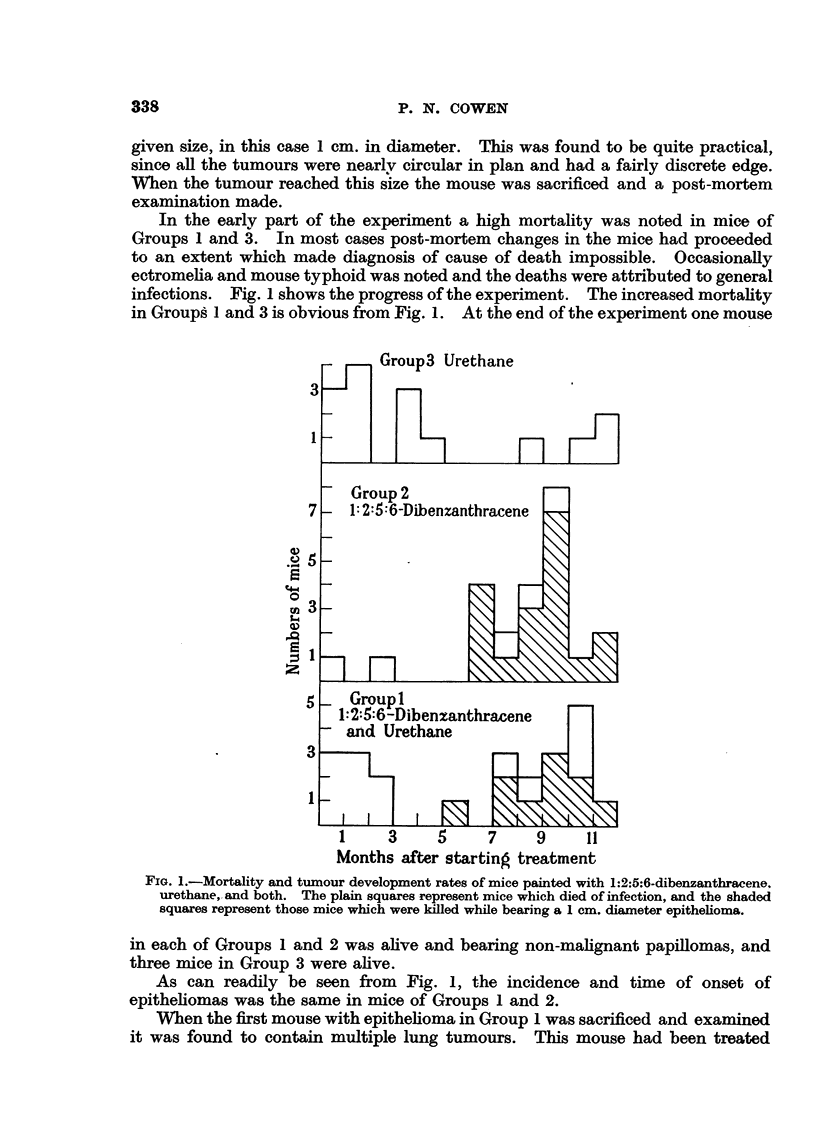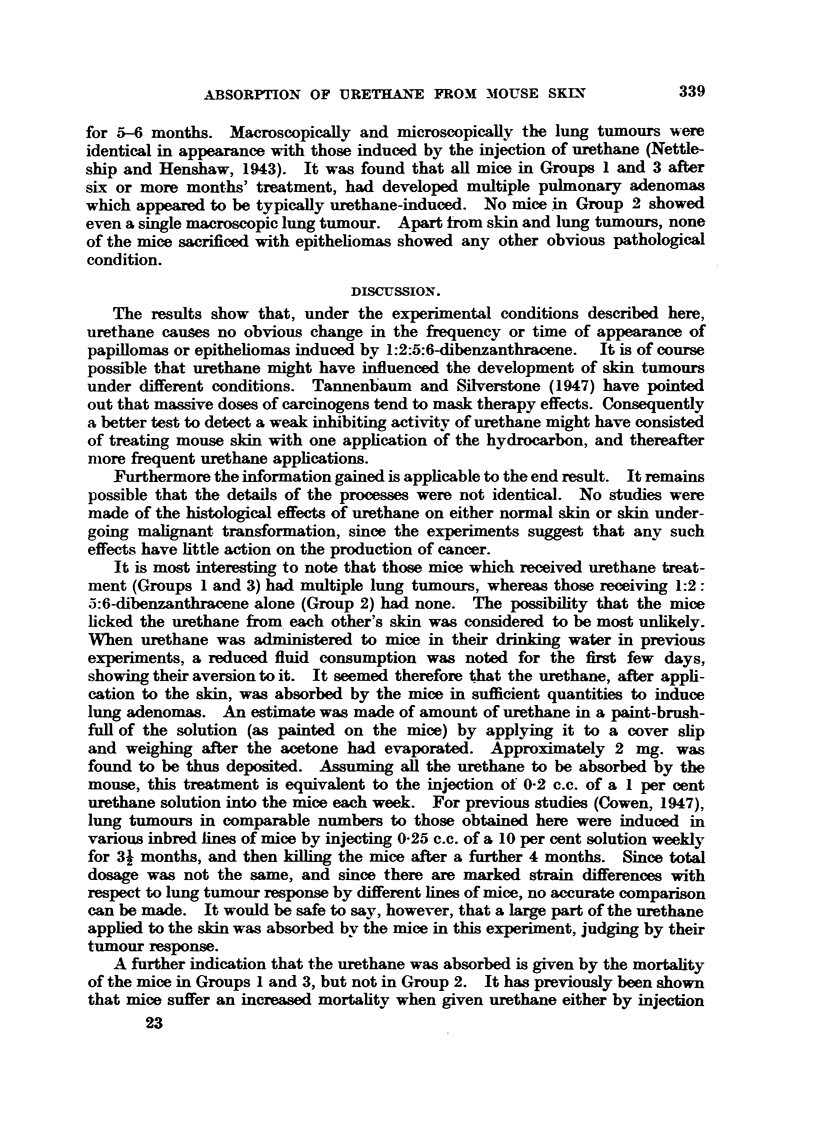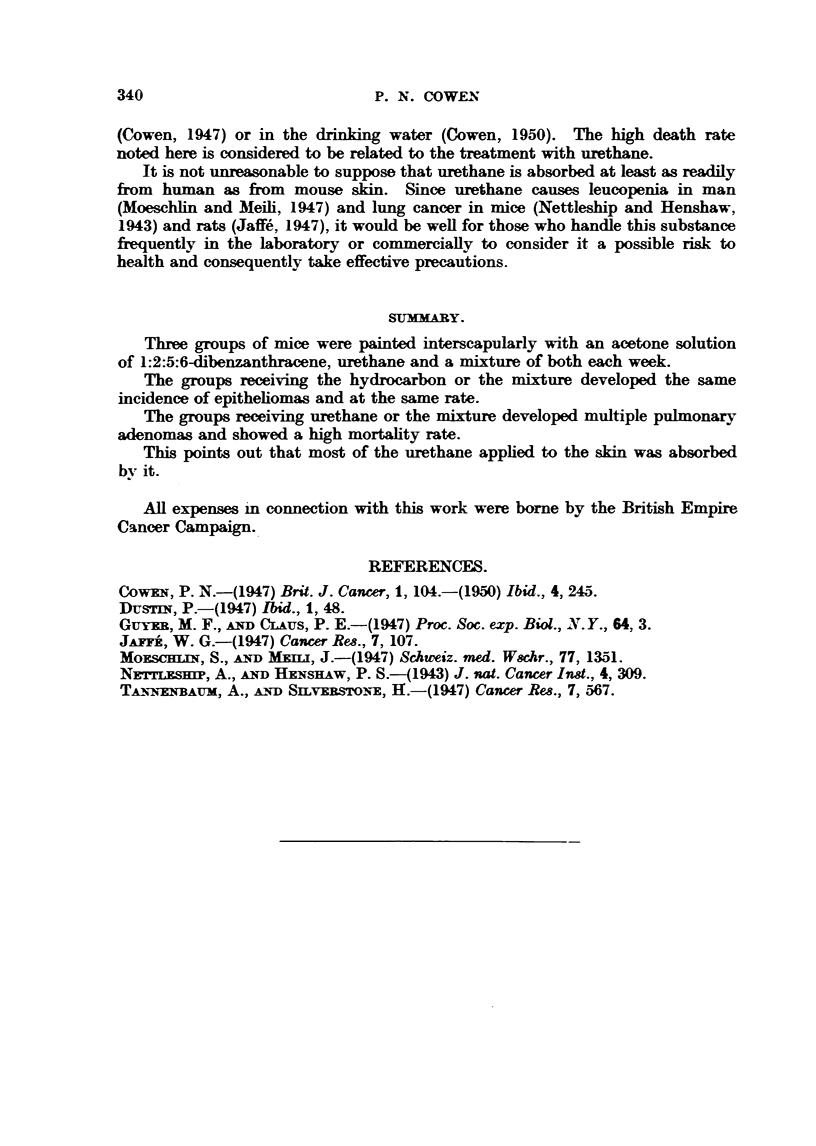# The Absorption of Urethane from Mouse Skin

**DOI:** 10.1038/bjc.1950.32

**Published:** 1950-09

**Authors:** P. N. Cowen


### THE ABSORPTION OF URETHANE FROM MOUSE SKIN.

P. N. COWEN.

*From the British Empire Cancer Campaign Unit, Royal Cancer Hospital, Chalfont St. Giles, Bucks.*

Received for publication September 4, 1950.

URETHANE causes a very marked depression of mitotic activity of certain mouse, rat and rabbit cells (Dustin, 1947 ; Guyer and Claus, 1947). The present work was undertaken to determine whether any such action would affect the process of malignant transformation as brought about by painting a carcinogenic hydrocarbon on mouse skin.

### MATERIAL AND METHODS.

The carcinogen used was 1:2:5:6-dibenzanthracene and the urethane was applied simultaneously. Young adult stock laboratory male mice were divided into three groups and treated with solutions as shown in Table I.

TABLE I.—*Number of Mice in Each Group and their Treatment.*

| Group. | Number of mice. | |
|---|---|---|
| 1 | 24 | 20 per cent urethane (w/v) in a saturated solution of 1:2:5:6-dibenzanthracene in acetone. |
| 2 | 24 | Saturated solution of 1:2:5:6-dibenzanthracene in acetone. |
| 3 | 18 | 20 per cent urethane in acetone (w/v). |

A drop of the solution was applied interscapularly on the skin of the mice, by paint-brush, once a week, for one year till the mice died or were sacrificed. In this way a local and simultaneous action of urethane and hydrocarbon on mouse skin was obtained (Group 1). Group 2 served as a control, receiving the hydrocarbon only. Group 3 was given urethane alone in order to observe any independent effects which it might cause over a long period of time.

### RESULTS.

Most of the mice in Groups 1 and 2 epilated at the site of application and developed papillomas which eventually became malignant. The time taken for these processes varied greatly between individual mice of both Groups 1 and 2, but no group difference was noted. It was difficult to determine exactly when a papilloma became malignant, but once the malignant process began, the epitheliomas grew at a fairly constant rate. The best criterion of response to treatment was considered to be the time taken for an epithelioma to reach a given size, in this case 1 cm. in diameter. This was found to be quite practical, since all the tumours were nearly circular in plan and had a fairly discrete edge. When the tumour reached this size the mouse was sacrificed and a post-mortem examination made.

In the early part of the experiment a high mortality was noted in mice of Groups 1 and 3. In most cases post-mortem changes in the mice had proceeded to an extent which made diagnosis of cause of death impossible. Occasionally ectromelia and mouse typhoid was noted and the deaths were attributed to general infections. Fig. 1 shows the progress of the experiment. The increased mortality in Groups 1 and 3 is obvious from Fig. 1. At the end of the experiment one mouse

FIG. 1.—Mortality and tumour development rates of mice painted with 1:2:5:6-dibenzanthracene, urethane, and both. The plain squares represent mice which died of infection, and the shaded squares represent those mice which were killed while bearing a 1 cm. diameter epithelioma.

in each of Groups 1 and 2 was alive and bearing non-malignant papillomas, and three mice in Group 3 were alive.

As can readily be seen from Fig. 1, the incidence and time of onset of epitheliomas was the same in mice of Groups 1 and 2.

When the first mouse with epithelioma in Group 1 was sacrificed and examined it was found to contain multiple lung tumours. This mouse had been treated for 5–6 months. Macroscopically and microscopically the lung tumours were identical in appearance with those induced by the injection of urethane (Nettleship and Henshaw, 1943). It was found that all mice in Groups 1 and 3 after six or more months' treatment, had developed multiple pulmonary adenomas which appeared to be typically urethane-induced. No mice in Group 2 showed even a single macroscopic lung tumour. Apart from skin and lung tumours, none of the mice sacrificed with epitheliomas showed any other obvious pathological condition.

### DISCUSSION.

The results show that, under the experimental conditions described here, urethane causes no obvious change in the frequency or time of appearance of papillomas or epitheliomas induced by 1:2:5:6-dibenzanthracene. It is of course possible that urethane might have influenced the development of skin tumours under different conditions. Tannenbaum and Silverstone (1947) have pointed out that massive doses of carcinogens tend to mask therapy effects. Consequently a better test to detect a weak inhibiting activity of urethane might have consisted of treating mouse skin with one application of the hydrocarbon, and thereafter more frequent urethane applications.

Furthermore the information gained is applicable to the end result. It remains possible that the details of the processes were not identical. No studies were made of the histological effects of urethane on either normal skin or skin undergoing malignant transformation, since the experiments suggest that any such effects have little action on the production of cancer.

It is most interesting to note that those mice which received urethane treatment (Groups 1 and 3) had multiple lung tumours, whereas those receiving 1:2:5:6-dibenzanthracene alone (Group 2) had none. The possibility that the mice licked the urethane from each other's skin was considered to be most unlikely. When urethane was administered to mice in their drinking water in previous experiments, a reduced fluid consumption was noted for the first few days, showing their aversion to it. It seemed therefore that the urethane, after application to the skin, was absorbed by the mice in sufficient quantities to induce lung adenomas. An estimate was made of amount of urethane in a paint-brushfull of the solution (as painted on the mice) by applying it to a cover slip and weighing after the acetone had evaporated. Approximately 2 mg. was found to be thus deposited. Assuming all the urethane to be absorbed by the mouse, this treatment is equivalent to the injection of 0·2 c.c. of a 1 per cent urethane solution into the mice each week. For previous studies (Cowen, 1947), lung tumours in comparable numbers to those obtained here were induced in various inbred lines of mice by injecting 0·25 c.c. of a 10 per cent solution weekly for 3½ months, and then killing the mice after a further 4 months. Since total dosage was not the same, and since there are marked strain differences with respect to lung tumour response by different lines of mice, no accurate comparison can be made. It would be safe to say, however, that a large part of the urethane applied to the skin was absorbed by the mice in this experiment, judging by their tumour response.

A further indication that the urethane was absorbed is given by the mortality of the mice in Groups 1 and 3, but not in Group 2. It has previously been shown that mice suffer an increased mortality when given urethane either by injection

(Cowen, 1947) or in the drinking water (Cowen, 1950). The high death rate noted here is considered to be related to the treatment with urethane.

It is not unreasonable to suppose that urethane is absorbed at least as readily from human as from mouse skin. Since urethane causes leucopenia in man (Moeschlin and Meili, 1947) and lung cancer in mice (Nettleship and Henshaw, 1943) and rats (Jaffé, 1947), it would be well for those who handle this substance frequently in the laboratory or commercially to consider it a possible risk to health and consequently take effective precautions.

### SUMMARY.

Three groups of mice were painted interscapularly with an acetone solution of 1:2:5:6-dibenzanthracene, urethane and a mixture of both each week.

The groups receiving the hydrocarbon or the mixture developed the same incidence of epitheliomas and at the same rate.

The groups receiving urethane or the mixture developed multiple pulmonary adenomas and showed a high mortality rate.

This points out that most of the urethane applied to the skin was absorbed by it.

All expenses in connection with this work were borne by the British Empire Cancer Campaign.

### REFERENCES.

Cowen, P. N.—(1947) *Brit. J. Cancer*, 1, 104.—(1950) *Ibid.*, **4**, 245.

Dustin, P.—(1947) *Ibid.*, 1, 48.

Guyer, M. F., and Claus, P. E.—(1947) *Proc. Soc. exp. Biol., N.Y.*, **64**, 3.

Jaffé, W. G.—(1947) *Cancer Res.*, 7, 107.

Moeschlin, S., and Meili, J.—(1947) *Schweiz. med. Wschr.*, 77, 1351.

Nettleship, A., and Henshaw, P. S.—(1943) *J. nat. Cancer Inst.*, **4**, 309.

Tannenbaum, A., and Silverstone, H.—(1947) *Cancer Res.*, 7, 567.